# Diagnostic Performance of Endoscopic and Microscopic Procedures for Identifying Different Middle Ear Structures and Remaining Disease in Patients with Chronic Otitis Media: A Prospective Cohort Study

**DOI:** 10.1371/journal.pone.0132890

**Published:** 2015-07-13

**Authors:** Farhad Farahani, Elnaz Shariatpanahi, Javane Jahanshahi, Jalal Poorolajal

**Affiliations:** 1 Department of Otolaryngology, School of Medicine, Hamadan University of Medical Sciences, Hamadan, Iran; 2 Modeling of Noncommunicable Diseases Research Center, Department of Epidemiology, School of Public Health, Hamadan University of Medical Sciences, Hamadan, Iran; University Health Network and University of Toronto, CANADA

## Abstract

**Background:**

The diagnostic performance of endoscopic and microscopic procedures for detecting diseases of the middle ear in patients with chronic otitis media (COM) has rarely been investigated. This study was conducted to compare the performance of these procedures for identifying middle ear structures and their associated diseases in COM patients.

**Methods:**

In this prospective cohort study, 58 patients with chronic COM, who were candidates for tympanoplasty with or without a mastoidectomy, were enrolled. Before the surgical intervention, the middle ear was examined via an operating microscope and then through an endoscope to identify the middle ear structures as well as diseases associated with the middle ear.

**Results:**

The patients were 15 years of age or older. The anatomical parts of the middle ear – the epitympanic, posterior mesotympanic, and hypotympanic structures – were more visible through an endoscope than through a microscope. In addition, the various segments of the mesotympanum, oval window, round window, and Eustachian tube were more visible via endoscopy. The post-operative endoscopic reevaluation of the middle ear revealed that a cholesteatoma had remained in four of 13 patients after surgery.

**Conclusion:**

According to the results of this study, in cases in which there is poor visibility with the operating microscope or the surgeon suspects remaining disease within the middle ear, endoscopy could be utilized to improve the evaluation of more hidden middle ear pits and structures, particularly if there is a potentially recrudescent pathology.

## Introduction

Tympanoplasty and mastoidectomy aided by microscopic techniques are accepted and routine methods for operating on middle ear structures in patients with chronic otitis media (COM) [1]. Despite its common application, using a microscope for these procedures is typically accompanied with limited visibility of various middle ear components including the hypotympanum, sinus tympani, and epitympanum, as well as the posterior part of the mesotympanum [2,3]. The ability to use both hands is one of the main advantages of this procedure, and the technique has improved the diagnostic performance of the procedure [4–7].

In addition to microscopy techniques, the application of flexible and rigid endoscopy has become usual for clinical evaluation of the structures of the middle ear. The assessment of these structures has been facilitated by endoscopy [7]. Comparing microscopic and endoscopic diagnostic approaches has revealed the superiority and feasibility of the latter method in evaluating middle ear pathological changes and structural abnormalities [8,9]. Some studies have reported successful surgeries of the middle ear including myringoplasty [10], surgery of the retraction pocket [11], stapedotomy [12], and removal of dermoid tumors of the Eustachian tubes[13] using the endoscopic approach. Because of some potential limitations of the endoscopic procedure including iatrogenic trauma, induced hyperthermia, and one-handed application, the use of this procedure is relatively uncommon in clinical settings [14].

Although the diagnostic performances of these two procedures have been widely assessed in various studies in different settings [15–17], to the best of our knowledge, there is insufficient evidence to compare the diagnostic performance of the procedures in COM patients. This study was conducted to compare the diagnostic performance of endoscopic and microscopic procedures in identifying the middle ear structures and associated diseases in patients with COM.

## Materials and Methods

This prospective cohort study was conducted in the Otorhinolaryngology Department of Besat Hospital, Hamadan, Iran, from October 2011 to September 2012 with 58 consecutive COM patients, who were candidates for various types of tympanoplasty with or without a mastoidectomy. The Research Council for Research of Hamadan University of Medical Sciences approved the study. Written consent was obtained from the patients or their parents.

**Fig 1:**
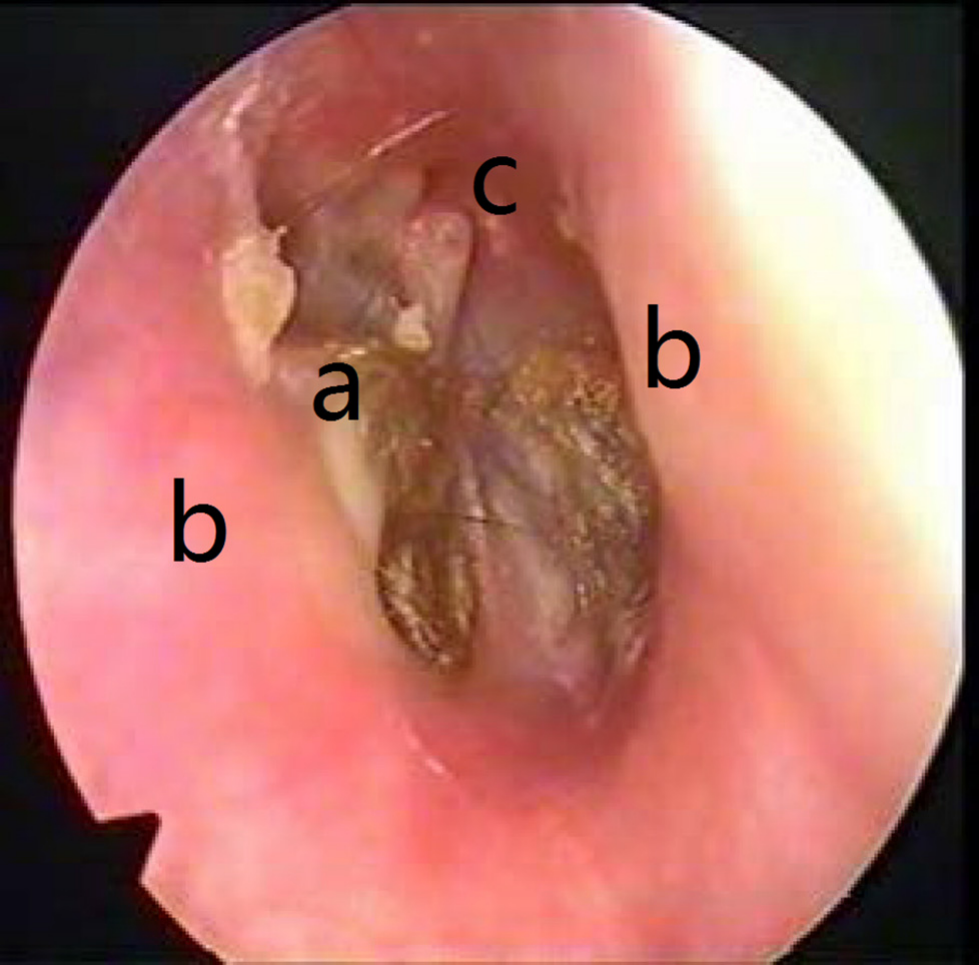
Preoperative transcanal microscopic view: (a) Cholesteatoma debris; (b) External auditory; canal bulging (obscures some part of the visual field); (c) Malleus.

**Fig 2:**
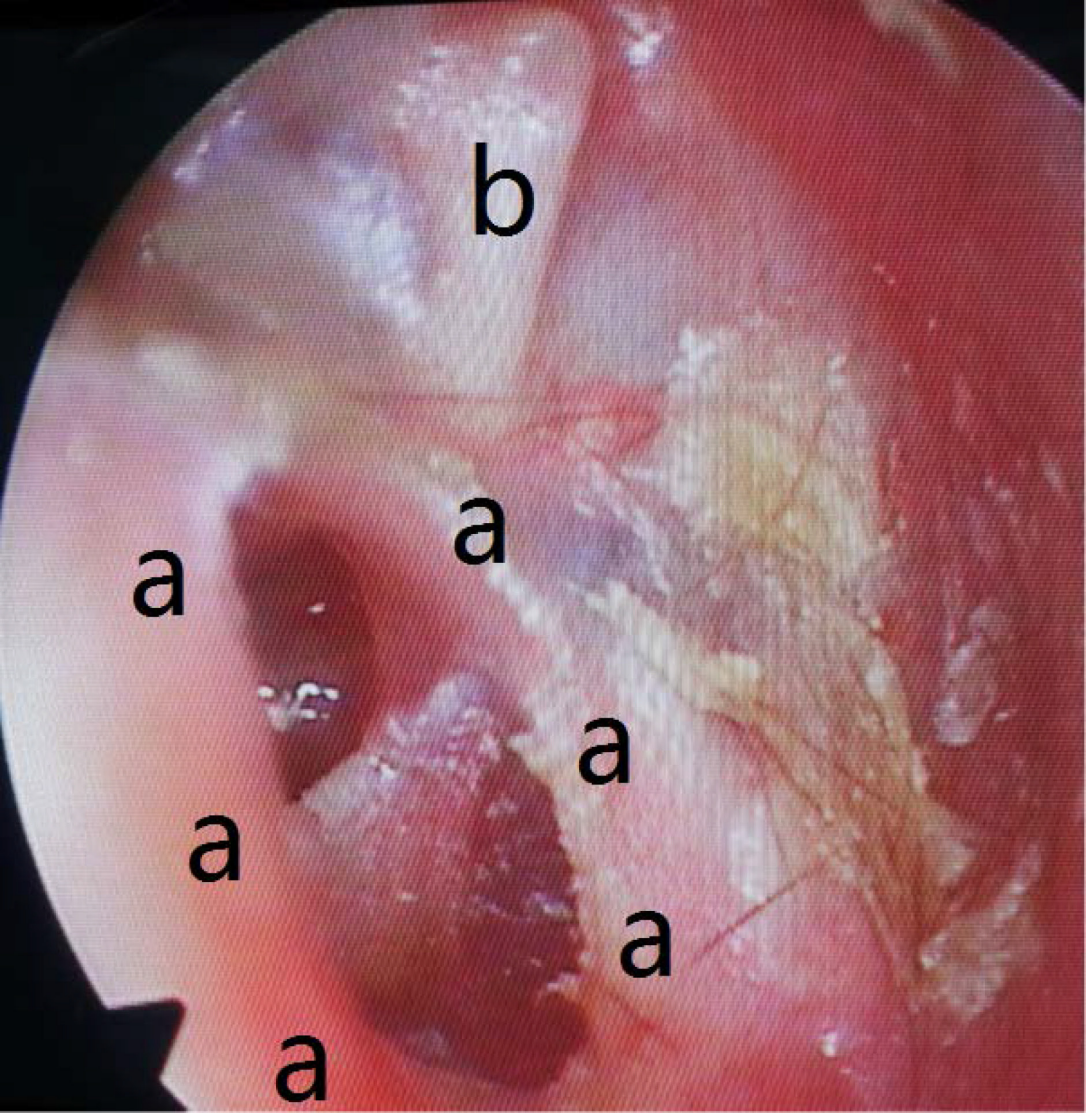
Preoperative transcanal endoscopic view: (a) Malleus; (b) Tympanic membrane perforation (evident after the cholesteatoma removal).

The baseline data on the demographic features, patients’ chief complaints (purulent discharge, hearing loss, or vestibular symptoms), duration of the disease, findings of the physical examination (Figs 1 and 2), results of tuning fork tests, and type of hearing loss (sensorineural, conductive or mixed) were collected. The candidates for second-stage hearing reconstruction or revision tympanoplasty with or without a mastoidectomy and the patients undergoing surgery by a non-post auricular incision were excluded from the study.

**Fig 3:**
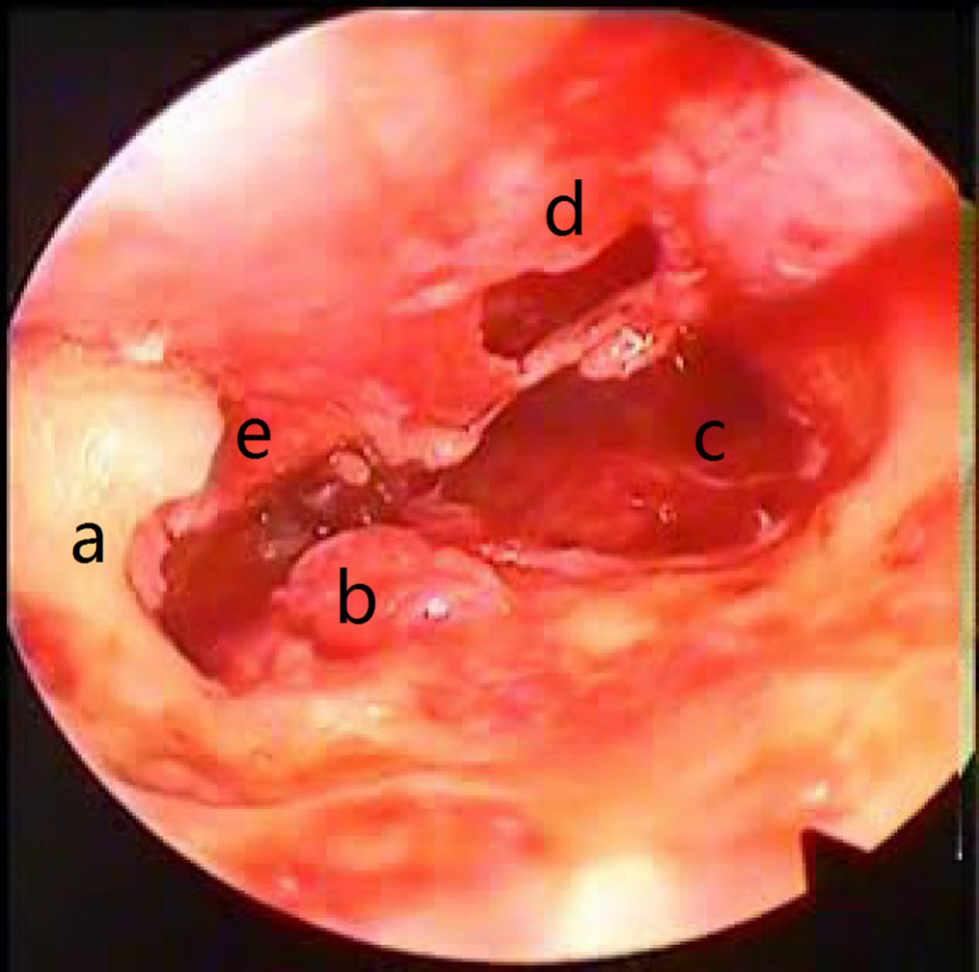
Preoperative middle ear microscopic view: (a) Scutum erosion; (b) Cholesteatoma and granulation tissue (around the stapes and facial recess); (d) Hypotympanum; (e) Tympanic membrane perforation; (f) Malleus.

**Fig 4:**
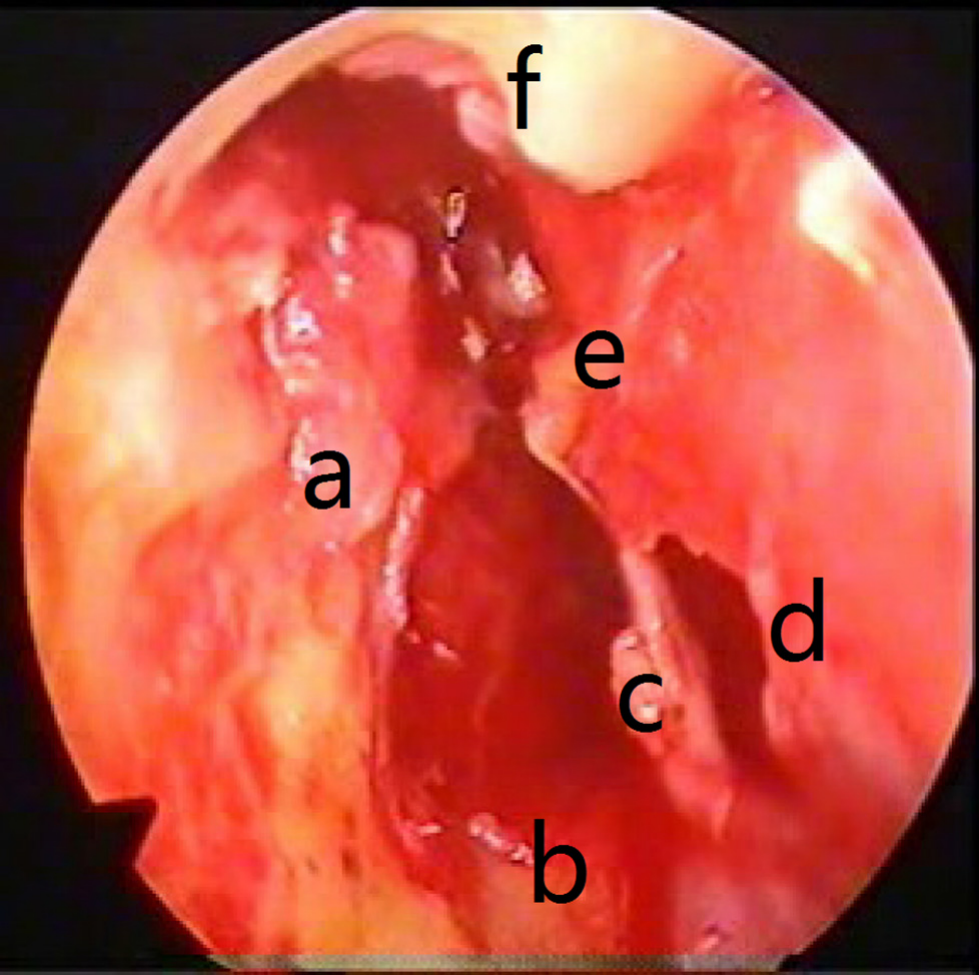
Preoperative middle ear endoscopic view: (a) Cholesteatoma and granulation tissue (around the stapes and facial recess); (b) Eustachian tube opening; (c) Hypotympanum; (d) Tympanic membrane perforation; (e) Malleus; (f) Scutum erosion.

Under general anesthesia, the middle ear was entered through a postauricular incision, and the tympanomeatal flap was elevated. Before the surgical intervention, the middle ear was examined with an operating microscope (Karl Storz, Germany with Sony 3CCD Color Video Camera, Japan) in different positions and in different bed positions. The visible anatomical areas were evaluated and recorded by performing gentle maneuvers on the patient`s head. Middle ear pathologies were explored with the identical technique, and the status of the ossicular chain was assessed as well. The middle ear was evaluated using a zero and 30-degree rigid endoscope (Karl Storz Image 1 HD H3 3-chip Camera Head and Diameter 4 mm, Work Length 18 cm, Karl Storz Image lens, Germany), and all of the components of the middle ear were assessed (Figs 3 and 4). The evaluations of the middle ear required approximately five minutes for each patient. Conventional middle ear surgery, using a microscope, was performed, and before the insertion of the tympanic membrane graft, the ear was reevaluated by endoscopy to detect any remaining disease. The exact anatomical sites were recorded, and a specimen was obtained for further pathological assessment, if any remaining disease were detected. Appropriate complementary surgical interventions were adapted for the effective eradication of detected pathologies. This second evaluation required approximately five minutes for each patient. The surgery was completed in a conventional method. All of the surgeries and microscopic and endoscopic evaluations were performed in real time by the same surgeon (a senior author, otologist and neuro-otologist, with 19 years of experience).

### Real time

After collecting the data, the statistical analysis was performed using SPSS software (version 15.0, SPSS, Inc., Chicago, Illinois). The categorical variables were compared using a chi-square test. P values of 0.006 were considered to be statistically significant based on the Bonferroni correction.

## Results

Included in the study were 58 consecutive patients, with an average age of 37.3 ±12.1 years (ranging from 15 to 63 years), 35 of whom were female. In 27 patients, the left ear was involved, and the right ear was involved in the others. Hearing loss, purulent discharge, and dizziness were the most common chief complaints of the patients. The average sensorineural hearing loss was 14.0 ±5.8 dB (ranging from 10 to 40), and the average conductive hearing loss was 31.9 ±9.3 dB (ranging from 15 to 50). The Rinne test was positive in 16 patients, and the Weber test was lateralized in 53 subjects.

The characteristics of the anatomical parts of the middle ear based on the microscopic and endoscopic findings are presented in Table 1. According to the results of this table, the epitympanum (P = 0.015) and posterior mesotympanum (P<0.001) structures as well as most parts of the mesotympanum were significantly more visible through the endoscope than through the microscope.

**Table 1:** The characteristics of the anatomical parts of the middle ear based on microscopic and endoscopic findings.

Structure	Microscope, n (%)	Endoscope, n (%)	*P* value ^a^
Epitympanum	18 (31.0)	31 (53.5)	0.015
Mesotympanum			
Malleus	47 (81.0)	47 (81.0)	1.000
Incus	39 (67.2)	40 (69.0)	0.842
Stapes	38 (65.5)	47 (81.0)	0.059
Oval window	33 (56.9)	46 (79.3)	0.010
Round window	39 (67.2)	52 (89.7)	0.003
Promontory	58 (100.0)	58 (100.0)	1.000
Eustachian tube	30 (51.7)	53 (91.4)	0.001
Facial nerve	3 (5.2)	3 (5.2)	1.000
Posterior mesotympanum			
Tympanic sinus	3 (5.2)	23 (39.7)	0.001
Hypotympanum	14 (24.1)	32 (55.1)	0.001

^a^ The P value of the Bonferroni correction for eight multiple testings = 0.006

As shown in Table 2, there was no significant difference in the evaluation of the ossicular chain mobility and the reflex of the round window between the microscopic and endoscopic approaches. In addition, according to the results presented in Table 3, the diagnostic performance of both procedures was similar in identifying ossicular chain erosions.

**Table 2:** The ossicular chain mobility and reflexes of the round window in the microscopic and endoscopic views.

Structure	Microscope		Endoscope		*P* value
	Good, n (%)	Reduced, n (%)	Good, n (%)	Reduced, n (%)	
Malleus mobility	42 (89.4)	5 (10.6)	42 (89.4)	5 (10.6)	1.000
Incus mobility	32 (82.0)	7 (18.0)	33 (82.5)	7 (17.5)	0.958
Stapes mobility	32 (82.2)	6 (17.8)	40 (85.1)	7 (14.9)	0.909
Round window reflex	31 (79.5)	8 (20.5)	41 (78.8)	9 (20.2)	0.765

**Table 3:** Ossicular chain erosion in the microscopic and endoscopic views.

Structure	Microscope		Endoscope		*P* value
	Healthy bone, n (%)	Erosive bone, n (%)	Healthy bone, n (%)	Erosive bone, n (%)	
Malleus erosion	47 (81.0)	11 (19.0)	47 (81.0)	11 (19.0)	1.000
Incus erosion	31 (51.4)	26 (44.8)	32 (55.1)	26 (44.8)	0.976
Stapes erosion	37 (63.8)	14 (24.1)	44 (75.9)	14 (24.1)	0.705

**Fig 5:**
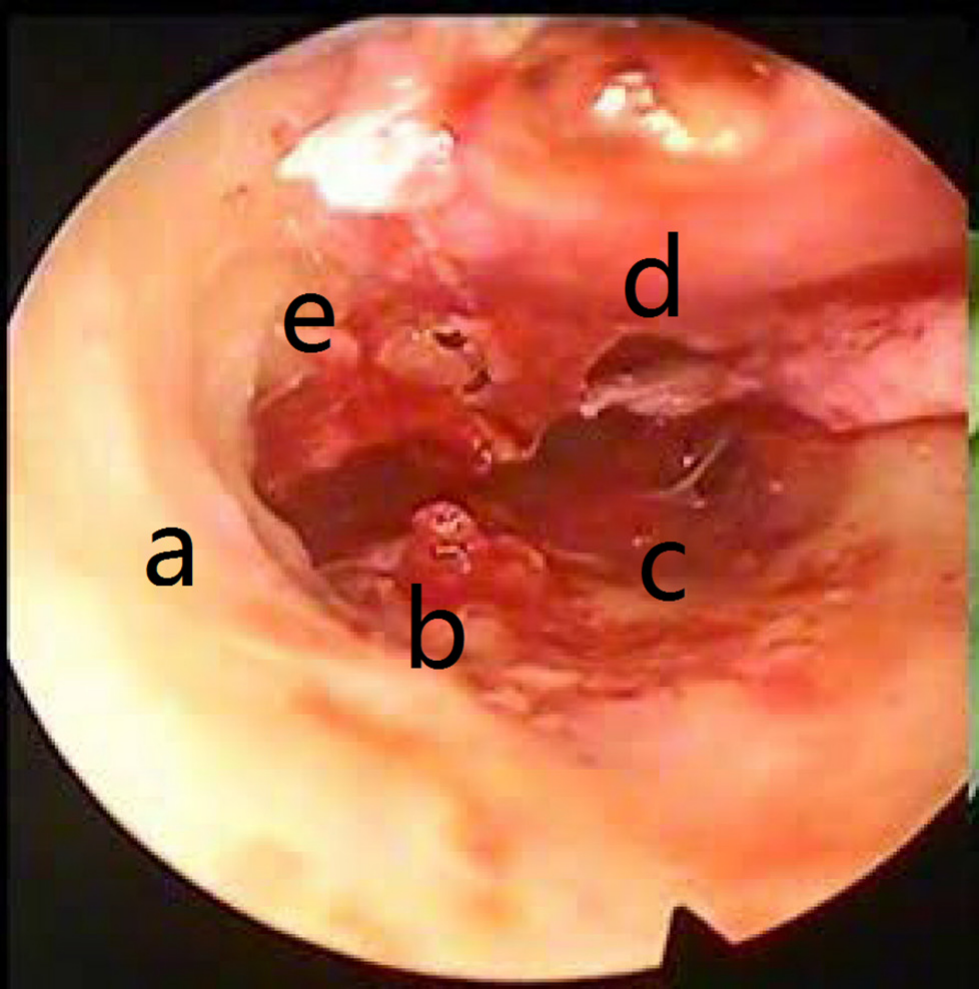
Postoperative middle ear microscopic view after the atticotomy and cholestetoma removal. (a) Scutum after the atticotomy; (b) Stapes capitalum; (c) Hypotympanic air cells; (d) Tympanic; (e) Membrane perforations.

**Fig 6:**
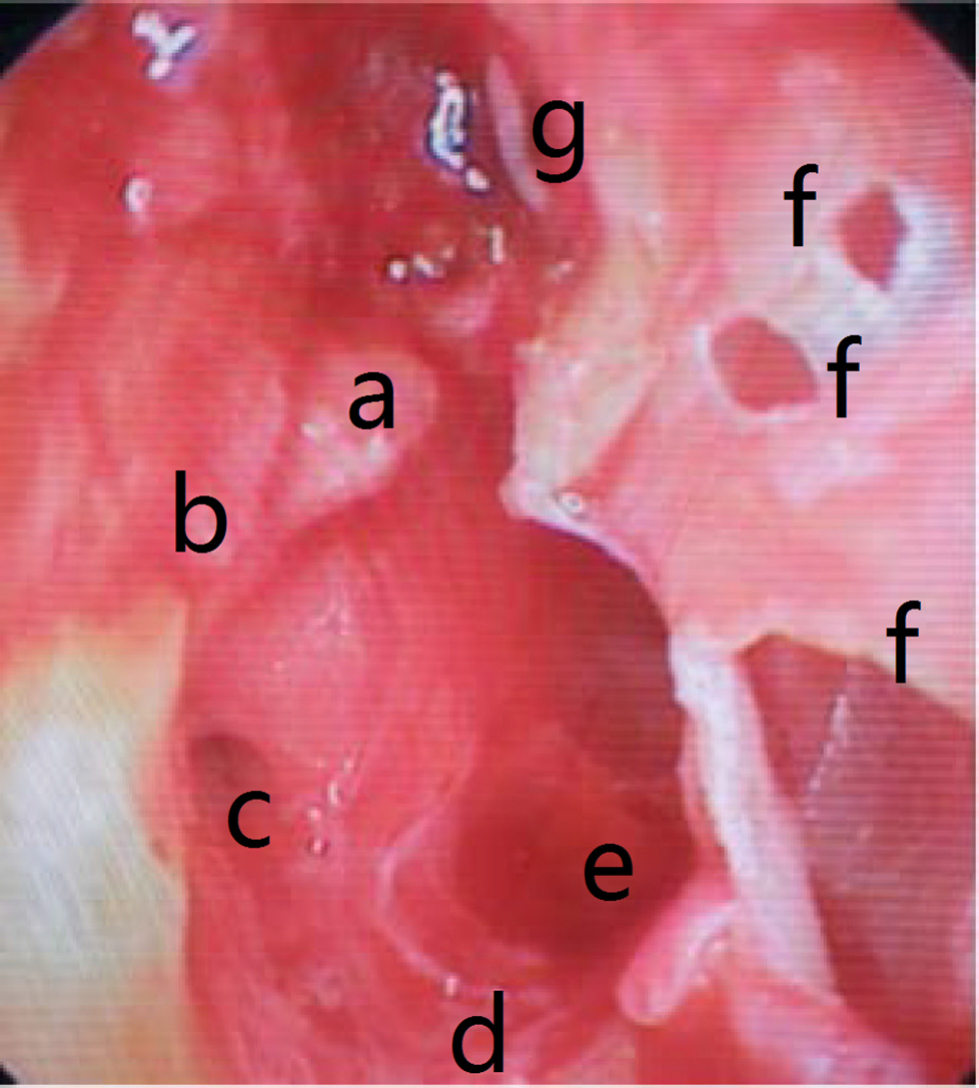
Postoperative middle ear endoscopic view (evidence of a minimal remaining cholesteatoma in the tympanic sinus). (a) Stapes capitalum; (b) The remaining cholesteatoma in the tympanic sinus; (c) Round window; niche; (d) Hypotympanic air cells; (e) Eustachian tube opening; (f) Tympanic membrane perforations; (g) Malleus.

At the end of the microscopic surgery and before the insertion of the tympanic membrane graft, the ear was reexamined with the endoscope to detect any remaining pathology. In 4 of the 13 patients with a cholesteatoma, the cholesteatoma had remained. This pathology was hidden in the sinus tympani in three patients and in the sinus tympani and epitympanum in another patient (Figs 5 and 6). In these patients, the surgery was continued for the effective eradication of the detected pathology. Granulation tissue was found in five patients. In one patient, it was detected in the medial surface of the scutum and could not be observed via microscope. Hypertrophic mucosa was found in 23 patients (most of them were in the hypotympanum), seven of which were detected by endoscopy. Tympanosclerotic plaque was found in 12 middle ears and could be seen through the microscope, except for one that was detected in the epitympanum. In two patients, there were polyps in the middle ear, both of which were observed via microscope. Other pathologies such as cholesterol granulomas were not detected.

## Discussion

To compare the diagnostic performance of microscopic and endoscopic approaches in COM patients, we examined these approaches to identify the pathological or structural abnormalities in different parts of the middle ear. The results of this study showed that both methods were comparable in viewing ossicular chain mobility and reflex of the round window as well as viewing ossicular chain erosions. Various anatomical parts of the middle ear, particularly the epitympanum, posterior mesotympanum, and hypotympanum, were more visible via endoscope than microscope. Some pathologies, such as cholesteatomas, are potentially recrudescent if they remain in the middle ear. A cholesteatoma had remained in four of 13 patients. These abnormalities were hidden in the sinus tympani more frequently than in other areas. In these patients, the surgery was continued to eradicate the detected cholesteatomas. Although several studies have assessed the diagnostic accuracy of microscopic and endoscopic procedures separately, to the best of our knowledge, this study was the first in which the visibility of the two approaches were compared for diagnosing abnormalities of the middle ear.

Most of the failures in the post auricular surgical approach have been associated with difficulties in viewing different pathologies in more hidden pits of the middle ear such as the epitympanum, posterior mesotympanum, and hypotympanum [18]. One of the most appropriate approaches to locating these pathologies is a stepwise trans canal assessment of the tympanic membrane and the middle ear cavity, followed by eradication of the probable pathologies [3]. Some studies have shown that the areas that are more visible by endoscopy are those whose pathologies are hidden, such as cholesteatomas [4,19]. These areas, in which the pathologies might be hidden, are the epitympanum, sinus tympani, and hypotympanum [1,3,7]. Because of these limitations, for many years, surgeons have sought better tools to improve the visibility of the middle ear [20–22]. Accordingly, the endoscopic approach to exploration of the middle ear was suggested. Because of its limitations in middle ear surgery, it has not been widely accepted, and microscopic surgery remains the first choice method for surgical interventions in middle ear diseases [4,5].

Using endoscopy in middle ear surgeries has some limitations including the necessity using one instead of two hands [2], the creation of significant heat in the middle ear [2], and trauma to the middle ear because of undesirable hand movements[7]. To avoid damaging the middle ear structures and increasing morbidity, it is recommended that surgeons should not use an endoscope instead of a microscope in every ear surgery. Endoscopy could be used efficiently in particular situations such as in cases in which remaining pathologies (e.g., cholesteatomas) are suspected or if the posterior canal wall limits visibility in the confirmation of ossicular chain integrity. Another limitation of this study was that the raw data were not reevaluated by independent observer through a video review for checking reliability of the results.

## Conclusion

In cases in which visibility by microscopy is disturbed and the surgeon suspects that pathologies remain in the middle ear, endoscopy could be utilized efficiently to improve the visibility and assessment of additional hidden middle ear pits and structures, particularly if there were a potentially recrudescent pathology.
